# Prevalence and risk factors of childhood allergic diseases in eight metropolitan cities in China: A multicenter study

**DOI:** 10.1186/1471-2458-11-437

**Published:** 2011-06-06

**Authors:** Fei Li, Yingchun Zhou, Shenghui Li, Fan Jiang, Xingming Jin, Chonghuai Yan, Ying Tian, Yiwen Zhang, Shilu Tong, Xiaoming Shen

**Affiliations:** 1 Department of Developmental and Behavioral Pediatrics, Shanghai Institute of Pediatric Translational Medicine, Shanghai Children's Medical Centre, Shanghai Jiaotong University, School of Medicine, Shanghai, China; 2 Shanghai Key Laboratory of Children's Environmental Health, Shanghai Jiaotong University, School of Medicine, Shanghai, China; 3 The Key Laboratory of Children's Environmental Health, Ministry of Education, China; 4 Department of Statistics and Actuarial Sciences, East China Normal University, Shanghai, China; 5 School of Public Health and Institute of Health and Biomedical Innovation, Queensland University of Technology, Queensland, 4000, Australia

## Abstract

**Background:**

Several studies conducted during the past two decades suggested increasing trend of childhood allergic diseases in China. However, few studies have provided detailed description of geographic variation and explored risk factors of these diseases. This study investigated the pattern and risk factors of asthma, allergic rhinitis and eczema in eight metropolitan cities in China.

**Methods:**

We conducted a cross-sectional survey during November-December 2005 in eight metropolitan cities in China. A total of 23791 children aged 6-13 years participated in this survey. Questions from the standard questionnaire of the International Study of Asthma and Allergies in Children (ISAAC) were used to examine the pattern of current asthma, allergic rhinitis and eczema. Logistic regression analyses were performed to assess the risk factors for childhood allergies.

**Results:**

The average prevalence of childhood asthma, allergic rhinitis and eczema across the eight cities was 3∙3% (95% Confidence interval (CI): 3∙1%, 3∙6%), 9∙8% (95% CI: 9∙4%, 10∙2%) and 5∙5% (95% CI: 5∙2%, 5∙8%), respectively. Factors related to lifestyle, mental health and socio-economic status were found to be associated with the prevalence of childhood allergies. These risk factors were unevenly distributed across cities and disproportionately affected the local prevalence.

**Conclusions:**

There was apparent geographic variation of childhood allergies in China. Socio-environmental factors had strong impacts on the prevalence of childhood allergies; but these impacts differed across regions. Thus public health policies should specifically target at the local risk factors for each individual area.

## Background

It is a significant and growing challenge to control and manage chronic allergic diseases worldwide. Approximately 20% of the world population suffers from allergic diseases which cause substantial health care burden [1]. For example, according to World Health Organization (WHO), 300 million people suffered from asthma and 255,000 died of asthma in 2005 [2].

Around the year 2000, the International Study of Asthma and Allergies in Children (ISAAC) was conducted across different regions of the world, which indicated that China's allergic problems could not be ignored [3]. However, their results only covered two cities in mainland China. In 1990 and 2000, two national surveys were conducted and showed that the prevalence of asthma among children 0-14 years old was 1% and 1.97% [4], respectively, suggesting an increasing trend of asthma. These implied that the socioenvironmental changes might directly or indirectly affect the prevalence rates of asthma and allergies. However, few data are available on the national pattern of prevalence and associated risk factors of childhood allergies in China.

A national survey on children's sleep was conducted in eight Chinese cities in 2005 [5]. Since this survey covered a range of childhood illnesses including asthma, allergic rhinitis and eczema, it offers a unique opportunity to examine the national trends of children's allergic diseases and their risk factors in China.

## Methods

### Sample

A cross-sectional survey was undertaken among children aged 6-13 years old in eight Chinese cities during November and December 2005, using a cluster-stratified sampling method. The eight cities included Shanghai, Guangzhou, Xi'an, Wuhan, Harbin, Chengdu, Hohhot and Urumqi. These were capital cities of provinces located in four different regions (Additional File 1, Figure S1) [6]. The reason for choosing capital cities was that, in China, these capital cities generally have high levels of health resources and public awareness about childhood allergic diseases, and doctors generally follow the national standard of clinical diagnosis. Therefore, the reporting of childhood allergic conditions and many of their risk factors would be more reliable. Three to ten districts were randomly selected from each city, and 1-2 elementary schools from each district. The number of districts was determined by the size of the city and the number of schools determined by the size of the district. The final sample comprised thirty districts and 42 schools in urban areas, and 9 districts and 13 schools in suburban areas. Of 23,791 children from 6 grades of the chosen schools, 22,018 (the response rate: 92∙5%) participated in the survey and returned completed questionnaires (Additional File 2, Figure S2).

### Data collection

The survey was conducted by the Key Laboratory of Children's Environmental Health in Shanghai, China. A national steering committee, comprised highly experienced paediatricians and epidemiologists from each survey centre, was established; a uniform research protocol was used by each centre; and formal training for the survey interviewers was provided. Each questionnaire was completed by a parent or guardian of a child after an informed consent form was signed. To ensure credibility and accuracy of the survey, we randomly selected 239 children for re-evaluation of responses one month after the first interview. 123 questionnaires for testing parallel consistency (ie, the consistency between the results when father and mother complete the same survey at the same time) and 116 questionnaires for assessing test-retest reliability were completed. The internal consistency of overall questionnaire was good (Cronbach's alpha's coefficient: 0.73). The parallel consistency between mother and father was represented by intro-class correlation coefficient (ICC: 0.89), and the test-retest reliability was also high (ICC: 0.85). To allow for comparison with results in the literature, we used key questions from the standard ISAAC questionnaire to examine the prevalences of current asthma, allergic rhinitis and eczema. The questions regarding asthma, allergic rhinitis and eczema were: "Has the child had asthma in the past 12 months?" (Yes or no), "Has the child had allergic rhinitis in the past 12 months?" (Yes or no) and "Has the child had eczema in the past 12 months?" (Yes or no). Data on a wide range of variables concerning demographic, lifestyle, mental health, socioeconomic status (SES) and health conditions were also collected: including child's age, gender, mode of delivery, breast feeding, diagnosed obesity, diagnosed gastroesophageal reflux (GER), carbonated drinks intake, computer use for amusements, schoolwork burden, mood, environmental tobacco exposure, parental alcohol abuse, parental smoking, maternal diagnosed depression during pregnancy and postpartum period, parental diagnosed depression, family structure, diagnosed childhood attention deficit hyperactivity disorder (ADHD), parental education level, household income per capita, resident area per capita, family size, common cold, diagnosed recurrent otitis media, snoring, sleep disordered breathing, parental snoring, parental sleep disordered breathing, sleep duration, and parental age at child's birth.

### Ethics statement

The ethical application of this study was approved by the local institutional review board at each research site, which included institutional review board of Shanghai Jiaotong University School of Medicine, Sichuan University West China Center of Medical Sciences, Sun Yat Sen University Medical School, Huazhong University of Science and Technology Tongji Medical University, Xi'an Jiaotong University College of Medicine, Harbin Medical University, Inner Mongolia Medical College and Xinjiang Medical University. We had obtained the written informed consent from all participants involved in our study.

### Statistical analysis

The prevalence rates of asthma, allergic rhinitis and eczema of each city were adjusted for age and sex [7], when compared across cities. Differences among cities were examined using chi-square tests for categorical variables and analyses of variance for continuous variables (significance level p = 0∙05 for two tails).

Logistic regression analyses were performed to assess the risk factors of allergies such as demographic variables, life styles, mental health and SES. Interaction terms of specific variables were also considered. The regression model adopted a forward stepwise elimination procedure, with probabilities for variable entry and removal of 0∙05 and 0∙10, respectively. Statistical tests of the regression estimates or odds ratios (OR) were based on Wald statistics. All analyses were conducted using SPSS version 13.0.

## Results

### Characteristics of study subjects

The mean age of the sample was 9∙18 years (SD = 1∙75), with 10,366 male students (49∙6%) and 10,548 female students (50∙4%). Of the 22018 children, the vast majority (20,719; 94∙1%) came from the Han ethnic group, with the remaining children (1,299; 5∙9%) from other ethnic minorities.

There was marked difference in some characteristics across cities. (Additional File 3, Table S1). For example, compared with those from other regions, participants from the eastern region (including Shanghai and Guangzhou) appeared to have higher levels of parental education and household income per capita, higher diagnosed obesity rates and more frequent computer use for amusement, lower vaginal delivery and breast feeding rates.

### Patterns of allergic diseases

**Figure 1:**
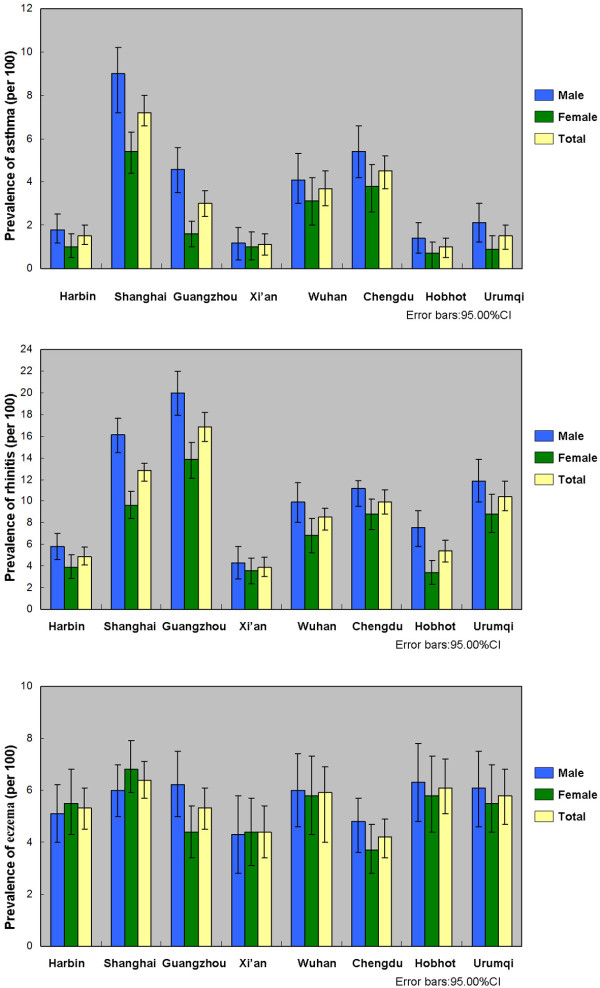
**Prevalence of asthma, allergic rhinitis and eczema by gender across cities**.

Table 1 shows both crude and adjusted (adjusted for age and sex) prevalences of three allergic diseases of each city. Figure 1 reveals the crude prevalence by gender across cities. The average prevalence of asthma, allergic rhinitis and eczema across eight cities was 3∙3% (3∙1, 3∙6), 9∙8% (9∙4, 10∙2) and 5∙5% (5∙2, 5∙8), respectively. There were statistically significant differences in the prevalences of asthma and allergic rhinitis (p < 0∙001) among the eight cities. There was no statistically significant difference in the geographic pattern of eczema.

**Table 1:** Crude and adjusted* prevalence rates (%) of three allergic diseases

		Asthma		Allergic rhinitis		Eczema	
		
	Number	Crude prevalence (%)(95% CI )	Adjusted prevalence (%)(95% CI )	Crude prevalence (%)(95% CI )	Adjusted prevalence (%)(95% CI )	Crude prevalence (%)(95% CI )	Adjusted prevalence (%)(95% CI )
**Total**	**22,009**	**3**∙**3 (3**∙**1-3**∙**6)**	**3**∙**3 (3**∙**1-3**∙**6)**	**9**∙**8 (9**∙**4-10**∙**2)**	**9**∙**8 (9**∙**4-10**∙**2)**	**5**∙**5 (5**∙**2-5**∙**8)**	**5**∙**5 (5**∙**2-5**∙**8)**
Harbin(4)	2,900	1∙4 (1∙0-1∙9)	1∙7 (1∙0-2∙4)	4∙9 (4∙1-5∙7)	4∙9 (3∙9-5∙8)	5∙3 (4∙5-6∙1)	4∙7 (3∙9-5∙5)
Shanghai(10)	4,395	7∙2 (6∙4-8∙0)	7∙0 (6∙0-7∙9)	12∙9 (11∙9-13∙9)	13∙1 (11∙7-14∙5)	6∙4 (5∙7-7∙1)	6∙5 (5∙4-7∙5)
Guangzhou(4)	3,094	3∙0 (2∙4-3∙6)	3∙0 (2∙4-3∙6)	16∙7 (15∙4-18∙0)	16∙8 (15∙3-18∙2)	5∙3 (4∙5-6∙1)	5∙4 (4∙6-6∙2)
Xi'an(3)	1,653	1∙1 (0∙6-1∙6)	1∙1 (0∙6-1∙7)	3∙9 (3∙0-4∙8)	3∙9 (2∙9-4∙9)	4∙4 (3∙4-5∙4)	4∙4 (3∙4-5∙4)
Wuhan(4)	2,061	3∙6 (2∙8-4∙4)	3∙5 (2∙7-4∙3)	8∙5 (7∙3-9∙7)	8∙3 (7∙1-9∙6)	6∙0 (5∙0-7∙0)	6∙1 (5∙0-7∙2)
Chengdu(5)	2,848	4∙5 (3∙8-5∙3)	4∙6 (3∙8-5∙4)	9∙9 (8∙8-11∙0)	10∙1 (8∙9-11∙2)	4∙2 (3∙5-4∙9)	4∙3 (3∙5-5∙1)
Hohhot(5)	2,025	1∙0 (0∙6-1∙5)	0∙9 (0∙5-1∙4)	5∙4 (4∙4-6∙4)	4∙5 (3∙6-5∙4)	6∙2 (5∙2-7∙2)	6∙4 (4∙9-7∙9)
Urumqi(4)	2,033	1∙5 (1∙0-2∙1)	1∙6 (1∙0-2∙1)	10∙3 (9∙0-11∙6)	10∙1 (8∙6-11∙5)	5∙9 (4∙9-6∙9)	5∙9 (4∙8-6∙9)

**P		<0∙001	<0∙001	<0∙001	<0∙001	<0∙001	<0∙001

Numbers of centers is indicated in brackets next to city name.*Adjusted to the age and gender distribution of the total sample. **P-value for difference between sites.

### Risk factors of allergic diseases

Table 2 shows the predictors of asthma, allergic rhinitis and eczema at the national level. In general, these allergies have many risk factors including *biological *(eg, mode of delivery, breast feeding, and sleep-disordered breathing); ***lifestyle ***(eg, diagnosed obesity, carbonated drinks intake, and frequent computer use as amusement); ***mental health ***(eg, diagnosed prepartum and postpartum depression, diagnosed childhood ADHD, and family structure); *SES *(eg, paternal education level, maternal education level, and household income per capita) and ***health conditions ***(eg, common cold, paternal snoring history, diagnosed recurrent otitis media, and diagnosed GER and snoring). Three allergic diseases seemed to share some common risk factors including residential city, diagnosed childhood ADHD, maternal education level, common cold, paternal snoring, and sleep-disordered breathing. Additionally, asthma and allergic rhinitis even shared more common risk factors such as gender, household income per capita, and paternal age at child's birth.

**Table 2:** Multiple logistic regression of risk factors for three allergic diseases

Variables		Asthma OR(95% CI )	Allergic rhinitis OR(95% CI )	Eczema OR(95% CI )
Gender	Male	**1**∙**5(1**∙**3-1**∙**8 )**	**1**∙**4 (1**∙**2-1**∙**5 )**	
Age^3^			**1**∙**1(1**∙**0-1**∙**2 )**	
Diagnosed GER	Yes	**2**∙**3(1**∙**2-4**∙**3)**		
Diagnosed obesity	Yes	**1**∙**7(1**∙**1-2**∙**7 )**		**1**∙**5 (1**∙**0-2**∙**3)**
Mode of delivery	Caesarean (Ref.)			
	Vaginal	**0**∙**8(0**∙**7-0**∙**9 )**		
Carbonated drinks intake	> = 5 times/week	**1**∙**8(1**∙**3-2**∙**6)**		
Exclusive breast feeding	> = 4 months		**0**∙**8(0**∙**7-0**∙**9)**	
Computer use as amusements	> = 5 times/week			**1**∙**3(1**∙**1-1**∙**6)**
Diagnosed childhood ADHD	Yes	**1**∙**4(1**∙**0-2**∙**0)**	**1**∙**7(1**∙**4-2**∙**1)**	**1**∙**5(1**∙**2-2**∙**0)**
Diagnosed prepartum and postpartum depression	Yes			**1**∙**7(1**∙**3-2**∙**4)**
Family structure	Single-parent and extended family (Ref.)			
	Nuclear family	**0**∙**8(0**∙**7-1**∙**0)**		
Household income per capita	> = 1500RMB/month	**1**∙**3(1**∙**1-1**∙**7)**	**1**∙**2(1**∙**1-1**∙**4)**	
Maternal education level	> = High school graduate	**1**∙**5(1**∙**2-1**∙**8)**	**1**∙**2(1**∙**1-1**∙**4)**	**1**∙**3(1**∙**2-1**∙**6)**
Paternal education level	> = High school graduate		**1**∙**3(1**∙**1-1**∙**4)**	
Maternal education level *Household income per capita	> = High school graduate and > = 1500RMB/month	**1.6(1.1,2.4)**		
Common cold	>5 times/year	**3**∙**2(2**∙**7-3**∙**8)**	**2**∙**3(2**∙**1-2**∙**6)**	**1**∙**8(1**∙**5-2**∙**1)**
Snoring	Yes		**1**∙**5(1**∙**3-1**∙**7)**	
Sleep-disordered breathing	Yes	**2**∙**3(1**∙**6-3**∙**1)**	**2**∙**3(1**∙**9-2**∙**9)**	**1**∙**5(1**∙**1-2**∙**0)**
Paternal snoring	Yes	**1**∙**2(1**∙**0-1**∙**5)**	**1**∙**2(1**∙**1-1**∙**3)**	**1**∙**2(1**∙**0-1**∙**4)**
Parental age at child's birth		**1**∙**2(1**∙**1-1**∙**3)**	**1**∙**1(1**∙**0-1**∙**3)**	
Diagnosed recurrent otitis media	Yes		**2**∙**1(1**∙**8-2**∙**6)**	**1**∙**9(1**∙**5-2**∙**4)**
Diagnosed asthma	Yes		**4**∙**3(3**∙**6-5**∙**2**)	**2**∙**7(2**∙**2-3**∙**4)**
Diagnosed allergic rhinitis	Yes	**3**∙**2(2**∙**7-3**∙**6)**		**1**∙**8(1**∙**5-2**∙**1)**
Diagnosed eczema	Yes	**2**∙**9(2**∙**3-3**∙**7**)	**1**∙**8(1**∙**5-2**∙**2)**	
Common cold*Snoring	Yes		**1.3(1.0,1.8)**	
Site	Urumqi (Ref.)			
	Shanghai	**4**∙**4(2**∙**9-6**∙**6)**	1∙0(0∙9-1∙3)	0∙9(0∙7-1∙2)
	Guangzhou	1∙4(0∙9-2∙2)	**1**∙**6(1**∙**3-1**∙**9)**	0∙8(0∙6-1∙1)
	Xi'an	**1**∙**4(3**∙**7-2**∙**6)**	**0**∙**4(0**∙**3-0**∙**6)**	0∙9(0∙6-1∙2)
	Wuhan	**2**∙**7(1**∙**7-4**∙**4)**	0∙9(0∙7-1∙1)	1∙1(0∙8-1∙5)
	Chengdu	**3**∙**2(2**∙**0-4**∙**9)**	0∙8(0∙7-1∙0)	0∙6(0∙5-0∙8)
	Harbin	1∙4(0∙8-2∙3)	**0**∙**5(0**∙**4-0**∙**6)**	1∙0(0∙7-1∙3)
	Hohhot	0∙9(0∙5-1∙7)	**0**∙**6(0**∙**4-0**∙**7)**	**1**∙**3(1**∙**0-1**∙**7)**

Values highlighted in bold indicate OR within 95% CI and statistically significant at P < 0∙05.

Tables 3, 4 and 5 show the specific risk factors of asthma, allergic rhinitis and eczema in the eight cities, respectively. Some cities shared common risk factors. For example, SES factors such as higher parental education level and higher household income per capita were associated with higher prevalences in Shanghai, Guangzhou, Wuhan and Chengdu, which are four biggest and affluent cities in this study. On the other hand, different cities had different spectrums of risk factors. For example, mental health factors were strongly associated with asthma in Wuhan (eg, OR = 3∙45, (95% CI: 1∙04, 11∙45) for diagnosed maternal depression), while life style factors such as diagnosed GER was a significant risk factor of asthma in Chengdu (OR = 14∙6, (95% CI: 12∙1, 41∙7), and 5∙3, (95% CI: 1∙2, 6∙7), respectively). Risk factors of allergic rhinitis (Table 4) and eczema (Table 5) show similar patterns as those of asthma (Table 3).

**Table 3:** Local level risk factors of asthma: results of multiple logistic regression analysis

Variables		Shanghai OR (95% CI )	Guangzhou OR (95% CI )	Xi'an OR (95% CI )	Wuhan OR (95% CI )	Chengdu OR (95% CI )	Harbin OR (95% CI )	Hohhot OR (95% CI )	Urumqi OR (95% CI )
Gender	Male	**1**∙**5** **(1**∙**1-1**∙**9)**	**2**∙**3** **(1**∙**4-3**∙**8)**						
Diagnosed GER	Yes					**14**∙**6** **(12**∙**1-41**∙**7)**		**10**∙**6** **(23**∙**4-48**∙**5)**	
Diagnosed Obesity	Yes						**14**∙**1** **(5**∙**0-40**∙**1)**	**7**∙**9** **(1**∙**7-35**∙**8)**	**7**∙**7** **(2**∙**2-27**∙**2)**
Carbonated drinks intake	> = 5 times/week					**5**∙**3** **(1**∙**2-6**∙**7)**			
Maternal diagnosed depression	Yes				**5**∙**5** **(1**∙**4-11**∙**4)**				
Diagnosed prepartum and postpartum depression	Yes	**2**∙**4** **(1**∙**2-4**∙**9)**							
Family structure	Single-parent and extended family (Ref.)								
	Nuclear family					**3**∙**2** **(1**∙**8-8**∙**5)**			
Household income per capita	> = 1500RMB/month		**2**∙**2** **(1**∙**1-1**∙**7)**		**1**∙**4** **(1**∙**1-3**∙**6)**				
Maternal education level	> = High school graduate	**1**∙**5** **(1**∙**2-2**∙**0)**	**1**∙**8** **(1**∙**1-3**∙**0)**						
Paternal education level	> = High school graduate					**2**∙**8** **(1**∙**8-4**∙**5)**			
Maternal education level *Household income per capita	> = High school graduate and > = 1500RMB/month		**1.6** **(1.0,2.7)**						
Paternal education level *Household income per capita	> = High school graduate and > = 1500RMB/month				**2.3** **(1.2,4.6)**				
Common cold	>5 times/year	**2**∙**6** **(1**∙**9-3**∙**3)**	**3**∙**8** **(2**∙**3-6**∙**1)**	**7**∙**1** **(2**∙**6-19**∙**2)**	**5**∙**8** **(3**∙**7-12**∙**3)**	**2**∙**7** **(1**∙**8-4**∙**2)**	**3**∙**9** **(2**∙**0-7**∙**8)**		**3**∙**0** **(1**∙**3-6**∙**7)**
Sleep-disordered breathing	Yes	**1**∙**9** **(1**∙**1-3**∙**4)**				**3**∙**9** **(1**∙**8-8**∙**5)**		**5**∙**3** **(1**∙**6-17**∙**1)**	**4**∙**1** **(1**∙**5-11**∙**4)**
Maternal snoring	Yes								**3**∙**3** **(1**∙**2-8**∙**7)**
Paternal age at child's birth		**1**∙**0** **(1**∙**0-1**∙**1)**							
Diagnosed allergic rhinitis	Yes	**5**∙**3** **(4**∙**0-6**∙**9)**	**4**∙**4** **(2**∙**7-7**∙**1)**		**4**∙**6** **(3**∙**0-10**∙**4)**	**5**∙**7** **(3**∙**7-8**∙**9)**	**3**∙**8** **(1**∙**6-9**∙**1)**	**5**∙**4** **(1**∙**8-16**∙**2)**	**2**∙**8** **(1**∙**1-6**∙**8)**
Diagnosed eczema	Yes	**3**∙**2** **(2**∙**2-4**∙**5)**	**2**∙**9** **(1**∙**5-5**∙**6)**		**2**∙**4** **(1**∙**1-5**∙**2)**	**2**∙**4** **(1**∙**2-4**∙**7)**	**4**∙**1** **(1**∙**8-9**∙**2)**		**3**∙**1** **(1**∙**1-8**∙**2)**

Values highlighted in bold indicate OR within 95% CI and statistically significant at P < 0∙05.

**Table 4:** Local level risk factors of allergic rhinitis: results of multiple logistic regression analysis

Variables		Shanghai OR (95% CI)	Guangzhou OR (95% CI)	Xi'an OR (95% CI)	Wuhan OR (95% CI)	Chengdu OR (95% CI)	Harbin OR (95% CI)	Hohhot OR (95% CI)	Urumqi OR (95% CI)
Gender	Female (Ref.)								
	Male	**1**∙**5** **(1**∙**3-1**∙**9)**	**1**∙**3** **(1**∙**1-1**∙**6)**		**1**∙**5** **(1**∙**0-2**∙**2)**	**2**∙**3** **(1**∙**7-3**∙**1)**		**2**∙**4** **(1**∙**5-3**∙**1)**	
Age		**1**∙**2** **(1**∙**1-1**∙**2)**			**1**∙**2** **(1**∙**1-1**∙**3)**			**1**∙**3** **(1**∙**1-1**∙**4)**	**1**∙**2** **(1**∙**1-1**∙**3)**
Exclusive breast feeding	> = 4 months	**0**∙**8** **(0**∙**6-0**∙**9)**			**0**∙**6** **(0**∙**4-0**∙**9)**				
Diagnosed childhood ADHD	Yes			**3**∙**1** **(1**∙**2-8**∙**2)**			**3**∙**4** **(1**∙**8-6**∙**2)**		
Household income per capita	> = 1500RMB/month	**1**∙**5** **(1**∙**1-2**∙**0)**				**1**∙**4** **(1**∙**1-1**∙**8)**	**1**∙**7** **(1**∙**1-2**∙**4)**		
Maternal education level	> = High school graduate	**1**∙**5** **(1**∙**2-1**∙**9)**						**2**∙**4** **(1**∙**6-3**∙**6)**	
Paternal education level	> = High school graduate				**1**∙**8** **(1**∙**2-2**∙**7)**				**1**∙**9** **(1**∙**4-2**∙**6)**
Paternal education level *Household income per capita	> = High school graduate and > = 1500RMB/month						**1.9** **(1.3,2.8)**		
Common cold	>5 times/year	**2**∙**3** **(1**∙**8-2**∙**8)**	**3**∙**2** **(2**∙**6-4**∙**0)**	**3**∙**5** **(1**∙**9-6**∙**6)**	**2**∙**7** **(1**∙**8-4**∙**1)**	**2**∙**3** **(1**∙**7-3**∙**1)**	**1**∙**6** **(1**∙**1-2**∙**6)**		**1**∙**7** **(1**∙**2-2**∙**4)**
Diagnosed recurrent otitis media	Yes	**2**∙**0** **(1**∙**3-3**∙**0)**	**2**∙**3** **(1**∙**6-3**∙**4)**	**5**∙**4** **(1**∙**9-16**∙**2)**	**3**∙**3** **(1**∙**7-6**∙**7)**	**2**∙**5** **(1**∙**6-3**∙**9)**	**3**∙**1** **(1**∙**6-5**∙**8)**		
Snoring	Yes	**1**∙**6** **(1**∙**2-2**∙**0)**	**1**∙**9** **(1**∙**4-2**∙**4)**		**2**∙**4** **(1**∙**5-3**∙**7)**		**1**∙**7** **(1**∙**0-2**∙**9)**		
Sleep-disordered breathing	Yes	**2**∙**4** **(1**∙**5-3**∙**9)**		**7**∙**0** **(2**∙**5-19**∙**6)**	**2**∙**3** **(1**∙**1-4**∙**6)**		**2**∙**9** **(1**∙**4-6**∙**2)**	**2**∙**3** **(1**∙**1-5**∙**1)**	
Paternal snoring	Yes		**1**∙**4** **(1**∙**1-1**∙**8)**						**1**∙**4** **(1**∙**0-1**∙**9)**
Maternal snoring	Yes							**2**∙**7** **(1**∙**3-5**∙**6)**	
Parental age at child's birth					**1**∙**1** **(1**∙**0-1**∙**3)**				
Maternal age at child's birth		**1**∙**0** **(1**∙**0-1**∙**1)**							
Diagnosed asthma	Yes	**5**∙**1** **(3**∙**9-6**∙**8)**	**3**∙**6** **(2**∙**2-5**∙**9)**		**4**∙**8** **(2**∙**5-9**∙**2)**	**5**∙**1** **(3**∙**4-1**∙**7)**	**3**∙**3** **(1**∙**3-8**∙**1)**	**6**∙**4** **(2**∙**2-18**∙**2)**	**2**∙**9** **(1**∙**2-6**∙**7)**
Diagnosed eczema	Yes	**1**∙**5** **(1**∙**1-2**∙**1)**	**1**∙**9** **(1**∙**3-2**∙**9)**			**2**∙**5** **(1**∙**5-4**∙**0)**		**3**∙**9** **(2**∙**2-6**∙**9)**	**1**∙**8** **(1**∙**1-3**∙**1)**
Common cold*Snoring	Yes	**2.4** **(1.4,3.9)**							

Values highlighted in bold indicate OR within 95% CI and statistically significant at P < 0∙05.

**Table 5:** Local level risk factors of eczema: results of multiple logistic regression analysis

Variables		Shanghai OR (95% CI )	Guangzhou OR (95% CI )	Xi'an OR (95% CI )	Wuhan OR (95% CI )	Chengdu OR (95% CI )	Harbin OR (95% CI )	Hohhot OR (95% CI )	Urumqi OR (95% CI )
Gender	Male	**0**∙**7** **(0**∙**5-0**∙**9)**							
Diagnosed Obesity	Yes	**2**∙**0** **(1**∙**1-3**∙**7)**							
Mode of delivery	Caesarean(Ref.)								
	Vaginal							**0**∙**6** **(0**∙**4-0**∙**7)**	
Carbonated drinks intake like Coke	> = 5 times/week							**2**∙**0** **(1**∙**1-3**∙**1)**	
Computer use as amusements	> = 5 times/week					**2**∙**0** **(1**∙**3-3**∙**3)**		**2**∙**1** **(1**∙**2-3**∙**9)**	
Paternal smoking	Yes			**1**∙**8** **(1**∙**0-3**∙**1)**					
Diagnosed childhood ADHD	Yes				**2**∙**2** **(1**∙**2-3**∙**9)**	**2**∙**7** **(1**∙**4-5**∙**3)**			
Paternal diagnosed depression	Yes				**2**∙**7** **(1**∙**5-6**∙**8)**				
Diagnosed prepartum and postpartum depression	Yes		**2**∙**4** **(1**∙**1-5**∙**8)**	**5**∙**9** **(2**∙**4-14**∙**6)**				**4**∙**7** **(2**∙**0-10**∙**8)**	
Overburdened schoolwork	Yes								**2**∙**0** **(1**∙**3-3**∙**0)**
Maternal education level	> = High school graduate	**1**∙**5** **(1**∙**1-1**∙**9)**	**1**∙**6** **(1**∙**1-2**∙**2)**				**1**∙**5** **(1**∙**1-2**∙**1)**		**2**∙**0** **(1**∙**3-3**∙**0)**
Paternal education level	> = High school graduate							**2**∙**1** **(1**∙**4-3**∙**2)**	
Common cold	>5 times/year	**2**∙**0** **(1**∙**5-2**∙**7)**		**3**∙**7** **(2**∙**0-6**∙**9)**	**2**∙**4** **(1**∙**5-3**∙**7)**		**2**∙**8** **(1**∙**9-4**∙**1)**	**1**∙**8** **(1**∙**1-3**∙**1)**	**3**∙**0** **(1**∙**9-4**∙**5)**
Snoring	Yes		**1**∙**6** **(1**∙**1-2**∙**3)**						
Sleep-disordered breathing	Yes	**1**∙**8** **(1**∙**0-3**∙**3)**							
Paternal snoring	Yes								**1**∙**7** **(1**∙**1-2**∙**5)**
Diagnosed recurrent otitis media	Yes	**2**∙**2** **(1**∙**4-3**∙**6)**				**2**∙**1** **(1**∙**1-4**∙**0)**			**3**∙**6** **(1**∙**6-7**∙**9)**
Diagnosed asthma	Yes	**3**∙**1** **(2**∙**2-4**∙**6)**	**2**∙**7** **(1**∙**4-5**∙**1)**	**4**∙**0** **(1**∙**0-15**∙**6)**		**2**∙**4** **(1**∙**3-4**∙**4)**	**3**∙**5** **(1**∙**5-7**∙**9)**	**6**∙**9** **(2**∙**4-2**∙**0)**	**3**∙**9** **(1**∙**6-9**∙**6)**
Diagnosed allergic rhinitis	Yes	**1**∙**5** **(1**∙**1-2**∙**1)**	**1**∙**9** **(1**∙**3-2**∙**8)**		**2**∙**1** **(1**∙**2-3**∙**7)**	**2**∙**4** **(1**∙**5-3**∙**9)**	**2**∙**1** **(1**∙**1-3**∙**8)**	**3**∙**2** **(1**∙**8-5**∙**7)**	

Values highlighted in bold indicate OR within 95% CI and statistically significant at P < 0∙05.

Because a stepwise selection procedure was used, only significant variables were included in the final models (Tables 3,4,5).

## Discussion

The present study reveals a geographic variation in the prevalences of allergic diseases across different regions of China. In particular, the regional variation in the prevalence of asthma was even wider than those in the US [8]. In general, the prevalence rates of allergic diseases were higher in more developed areas than poor areas.

The spectrum of risk factors for allergies in China had some common features with that in developed countries. Firstly, a cluster of unhealthy lifestyle factors which are widespread in developed countries such as diagnosed obesity, frequent use of computer for amusement (indicating physical inactivity) and excessive carbonated drinks intake (indicating unhealthy diets) were also important risk factors of childhood allergies in China [9-11]. Secondly, we found that a series of mental-health-related factors such as childhood ADHD, prepartum and postpartum depression, parental depression, non-nuclear family structure including single-parental or extended family and overburdened schoolwork were highly associated with the prevalences of childhood allergic diseases at the individual, household and school level.

However, some social determinants seem to have played more important roles in the occurrence of asthma in China than in developed countries. For example, prolonged exclusive breast-feeding (OR_allergic rhinitis_: 0∙84; 95% CI: 0∙76, 0∙93) and vaginal delivery (OR_asthma_: 0∙82; 95% CI: 0∙69, 0∙98) were both protective factors for allergies. Nevertheless in China the rates of these factors are markedly low. China's recently updated national rate of "exclusive breast feeding for six months" reached 21∙6%, which was far below the WHO target of 100% [12-14]. On the other hand, a recent WHO survey reveals that China had the world's highest rate of caesarean sections (up to 46∙2%), far exceeding the recommended upper threshold by WHO (15%)[15]. More concerning is that, among all the observed caesarean cases, 25% were done without medical indications, of which 62% were caused by financial incentives of hospitals [16].

Another striking difference between China and developed countries is the effects of mental health problems on allergic diseases: the stressors that were often found in developed countries at community or neighborhood level such as frequent exposure to violence and crime due to existence of residential segregation were rarely found in China[17]. On the other hand, stressors from the school, such as overburdened schoolwork detected in our study (OR_eczema_: 1∙96; 95% CI: 1∙27, 2∙98) (Table 5), have become more worrisome in China, since a highly competitive educational system had forced children to be exposed to high levels of stress from the start of primary school, which had caused lots of childhood psychosomatic symptoms [18].

A third difference is the effect of SES represented by parental education and household income per capita. The present study found that higher individual-level SES predicted higher prevalence of allergic diseases in China, in contrast to the findings from the developed countries that higher SES predicted lower prevalence[17], suggesting different hypothesis for explaining the impact of SES on allergies between China and developed countries.

Considering the local pattern of childhood allergies, some specific determinants influenced by social, cultural, and institutional structures were significantly associated with the local prevalence of allergic diseases. For example, psychological risk factors seem to have a considerable impact on the prevalence of asthma in Wuhan (Table 3). In fact, the proportion of childhood ADHD, prepartum and postpartum depression, and parental anxiety and depression in Wuhan were among the highest in the eight cities (Table 3), and all these were major risk factors of allergies. In Chengdu, some factors related to unhealthy dietary habits were found to be key risk factors of allergies, such as diagnosed GER (OR: 14∙6; 95% CI: 12∙1-41∙7). Chengdu is well-known for its historic food culture featuring heavy-oiled hot pot. It is widely believed that heavy-oiled hot pot is associated with GER, which is a leading risk factor of asthma in Chengdu.

Further investigation of the difference among cities using multi-level models found a significant association between childhood allergies and city-level socio-environmental factors. For example, GDP, PM10 and average humidity were strongly associated with childhood asthma. These will be discussed in a separate paper.

There are several limitations of the present study. Firstly, all the sampling sites were cities, thus only representing urban/suburban areas. Secondly, because this study was initially designed to address the issue of childhood sleep, there were some important allergic risk factors that were not included, for example, family history of asthma and allergies, and indoor and outdoor allergens.

## Conclusions

The present study found strong evidence of the connection between social, behavioural and biological factors and allergic diseases in China, indicating that strategies to reduce exposure to risk factors for childhood allergies could be broadened. In addition, local policies should specifically target at the risk factors for each individual area.

## Competing interests

The authors declare that they have no competing interests.

## Authors' contributions

This study was planned and implemented by XMS and ST, who are the principal investigators. XMS was responsible for the study's overall conception and design, acquisition of funding, supervision of data collection, and advice on clinical diagnostic methods. ST was responsible for the study's design and advice on epidemiological methods, data interpretation, critical revisions of the manuscript for important intellectual content, FL contributed to the conception and design of the study, analysis and interpretation of data, critical revisions of the manuscript for important intellectual content, and clinical diagnostic expertise for the study. YZ contributed to the analysis and interpretation of data, drafting of the manuscript, and epidemiological expertise for the study. SL, FJ, XJ, CY, YT and YZ implemented the study and offered critical revisions of the manuscript for important intellectual content. All authors read and approved the final manuscript.

## Pre-publication history

The pre-publication history for this paper can be accessed here:

http://www.biomedcentral.com/1471-2458/11/437/prepub

## Supplementary Material

Additional File 1 **Figure S1- Location of the sampling cities**. The eight cities were: Shanghai, Guangzhou, Xi'an, Wuhan, Harbin, Chengdu, Hohhot and Urumqi. These were capital cities of provinces located in four different regions cited from the 2006 China Statistical Yearbook according to their geographic locations, economic standards, and population densities. Click here for file

Additional File 2 **Figure S2-Samples and the sampling cities**. These sampling cities were capital cities of provinces located in four different regions cited from the 2006 China Statistical Yearbook according to their geographic locations, economic standards, and population densities. Three to ten districts were randomly selected from each city, and 1-2 elementary schools from each district. The selection was proportional to size so the number of districts was determined by the sizes of the cities and the number of schools determined by the sizes of the districts. The final sample comprised thirty districts and 42 schools in urban areas, and 9 districts and 13 schools in suburban areas. Click here for file

Additional File 3 **TableS1 - Characteristics of study subjects**. A wide range of variables concerning demographic, lifestyle, mental health, socioeconomic status and health conditions were also covered in this table. Click here for file
