# Supplementary figures and images for: Prevalence and risk factors of childhood allergic diseases in eight metropolitan cities in China: A multicenter study
Source: BMC Public Health. 2011 Jun 6;11:437. doi: 10.1186/1471-2458-11-437 (PMC3148998; doi:10.1186/1471-2458-11-437)

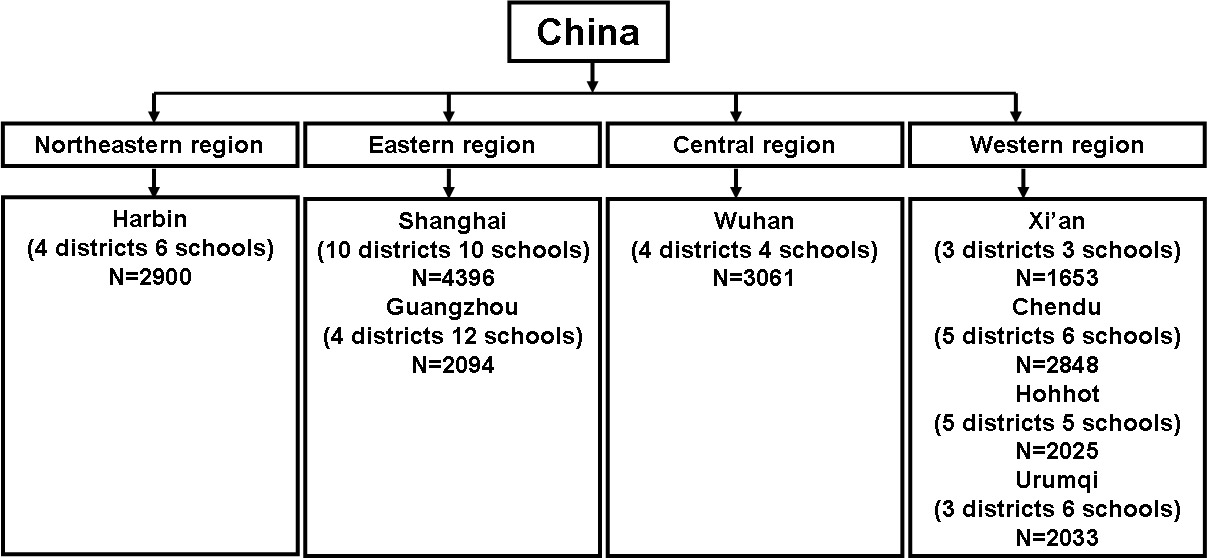

Supplement: Additional File 1 — Figure S1- Location of the sampling cities. The eight cities were: Shanghai, Guangzhou, Xi'an, Wuhan, Harbin, Chengdu, Hohhot and Urumqi. These were capital cities of provinces located in four different regions cited from the 2006 China Statistical Yearbook according to their geographic locations, economic standards, and population densities. [file 1471-2458-11-437-S1.JPEG]

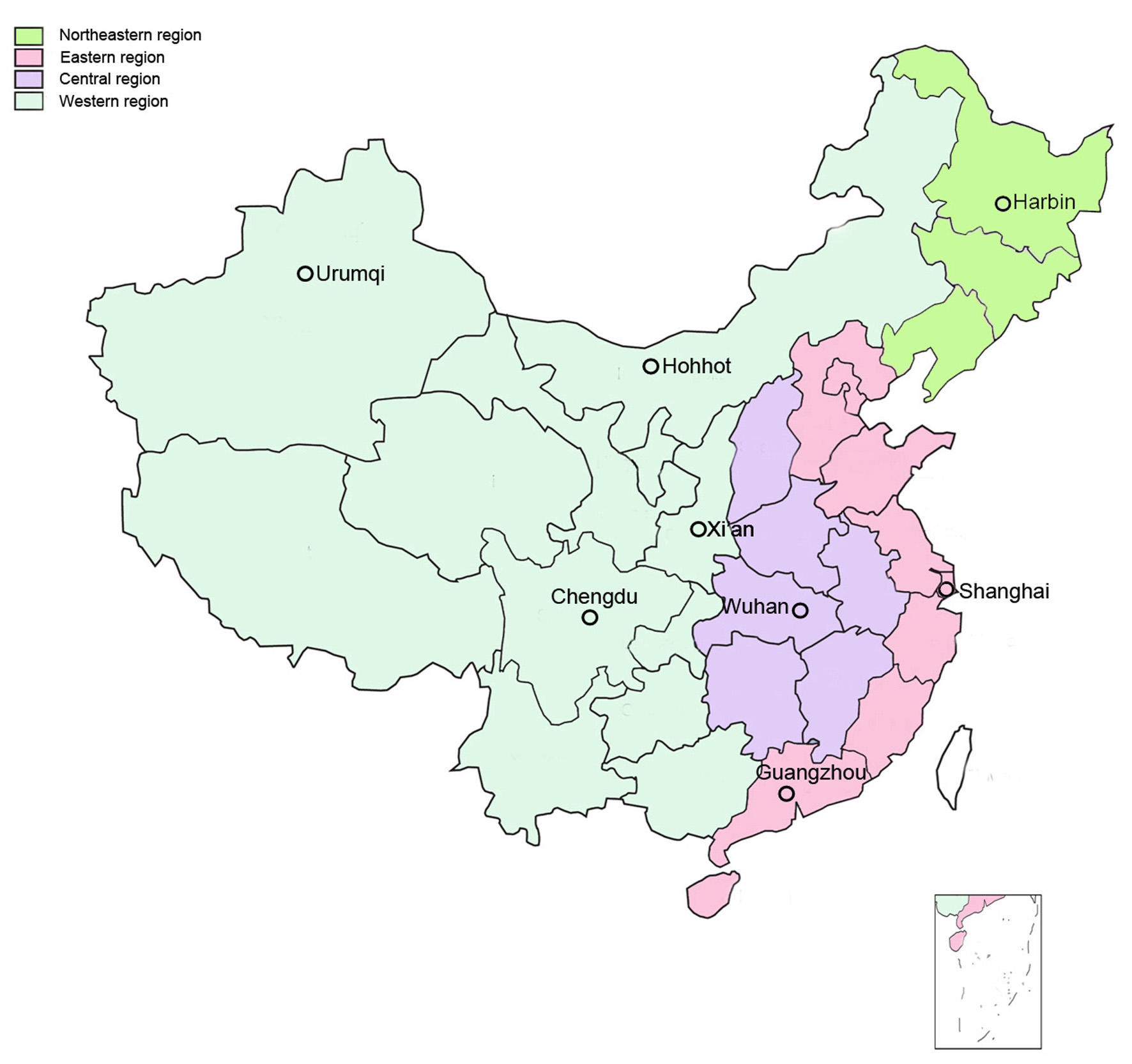

Supplement: Additional File 2 — Figure S2-Samples and the sampling cities. These sampling cities were capital cities of provinces located in four different regions cited from the 2006 China Statistical Yearbook according to their geographic locations, economic standards, and population densities. Three to ten districts were randomly selected from each city, and 1-2 elementary schools from each district. The selection was proportional to size so the number of districts was determined by the sizes of the cities and the number of schools determined by the sizes of the districts. The final sample comprised thirty districts and 42 schools in urban areas, and 9 districts and 13 schools in suburban areas. [file 1471-2458-11-437-S2.JPEG]
