# Supplementary material for: Prevalence and risk factors of childhood allergic diseases in eight metropolitan cities in China: A multicenter study
Source: BMC Public Health. 2011 Jun 6;11:437. doi: 10.1186/1471-2458-11-437 (PMC3148998; doi:10.1186/1471-2458-11-437)
Supplement: Additional File 3 — TableS1 - Characteristics of study subjects. A wide range of variables concerning demographic, lifestyle, mental health, socioeconomic status and health conditions were also covered in this table. [file 1471-2458-11-437-S3.DOC]

**Table S1*:* Characteristics of study subjects**

|  | **Shanghai** | **Guangzhou** | **Xi’an** | **Wuhan** | **Chengdu** | **Harbin** | **Hobhot** | **Urumqi** | **Total** |
| --- | --- | --- | --- | --- | --- | --- | --- | --- | --- |
| Number | 4395 | 3094 | 1653 | 2061 | 2848 | 2900 | 2025 | 2033 | 22009 |
| Male (%) | 49∙8 | 48∙4 | 42∙1 | 54∙5 | 46∙4 | 52∙4 | 49∙9 | 51∙9 | 49∙6 |
| Age (mean±SD) years | 8∙8±1∙49 | 9∙4±1∙84 | 9∙3±1∙83 | 9∙0±1∙80 | 9∙3±1∙84 | 8∙7±1∙52 | 10∙0±1∙82 | 9∙5±1∙80 | 9∙2±1∙77 |
| Mode of delivery (%) | | | | | | | | | |
| Vaginal (%) | 54∙1 | 65∙3 | 81∙0 | 81∙4 | 64∙2 | 60∙9 | 82∙4 | 71∙8 | 67∙3 |
| Caesarean (%) | 45∙9 | 34∙7 | 19∙0 | 18∙6 | 35∙8 | 39∙1 | 17∙6 | 28∙2 | 32∙7 |
| Exclusive breast feeding (%) | | | | | | | | | |
| <4 months (%) | 37∙9 | 42∙ 1 | 28∙1 | 24∙2 | 39∙2 | 32 | 25∙9 | 36∙5 | 34∙5 |
| >=4 months (%) | 62∙1 | 57∙9 | 71∙9 | 75∙8 | 60∙8 | 68∙0 | 74∙1 | 63∙5 | 65∙5 |
| Computer use as amusements (%) | | | | | | | | | |
| >=5 times/week (%) | 11∙7 | 13∙6 | 7∙4 | 6∙8 | 12∙5 | 10∙7 | 6∙4 | 10∙6 | 10∙5 |
| <5 times/week (%) | 88∙3 | 86∙4 | 92∙6 | 93∙2 | 87∙5 | 89∙3 | 93∙6 | 89∙4 | 89∙5 |
| Diagnosed Obesity (%) | | | | | | | | | |
| Yes (%) | 3∙1 | 2∙0 | 1∙2 | 1∙0 | 1∙5 | 1∙8 | 1∙1 | 1∙6 | 1∙8 |
| Diagnosed GER (%) | | | | | | | | | |
| Yes (%) | 0∙8 | 0∙5 | 0∙4 | 1∙4 | 0∙9 | 0∙4 | 1∙2 | 0∙9 | 0∙8 |
| Caffeinated drinks intake (such as coffee, tea and regular cola et al.) (%) | | | | | | | | | |
| >=5 times/week (%) | 3∙1 | 4∙2 | 4∙1 | 6∙0 | 4∙3 | 4∙2 | 7∙9 | 5∙9 | 4∙7 |
| <5 times/week (%) | 96∙9 | 95∙8 | 95∙9 | 94∙0 | 95∙7 | 95∙8 | 92∙1 | 94∙1 | 95∙3 |
| Environmental tobacco exposure (%) | | | | | | | | | |
| Yes (%) | 21∙0 | 30∙9 | 33∙5 | 18∙8 | 29∙4 | 23∙6 | 24∙0 | 20∙5 | 25∙0 |
| Paternal smoking (ex-smoker and current smoker) (%) | | | | | | | | | |
| Yes (%) | 58∙5 | 51∙0 | 62∙6 | 54∙4 | 58∙5 | 54∙1 | 57∙4 | 61∙7 | 56∙9 |
| Diagnosed prepartum and postpartum depression (%) | | | | | | | | | |
| Yes (%) | 1∙7 | 1∙9 | 3∙0 | 2∙7 | 3∙3 | 3∙3 | 3∙2 | 4∙2 | 2∙8 |
| Maternal diagnosed depression (%) | | | | | | | | | |
| Yes (%) | 1∙5 | 1∙5 | 2∙6 | 3∙1 | 2∙8 | 2∙1 | 2∙7 | 2∙4 | 2∙2 |
| Paternal diagnosed depression (%) | | | | | | | | | |
| Yes (%) | 0∙7 | 1∙0 | 1∙6 | 2∙6 | 2∙1 | 1∙1 | 1∙2 | 2∙2 | 1∙4 |
| Overburdened schoolwork (%) | | | | | | | | | |
| Yes (%) | 43∙9 | 39∙8 | 21∙0 | 29∙0 | 37∙2 | 24∙5 | 23∙8 | 25∙5 | 32∙7 |
| Family structure (%) | | | | | | | | | |
| Nuclear family (%) | 60∙9 | 59∙9 | 63∙2 | 56∙0 | 61∙4 | 64∙5 | 72∙9 | 69∙3 | 63∙0 |
| Single-parent and extended family (%) | 39∙1 | 40∙1 | 36∙8 | 44∙0 | 35∙5 | 35∙5 | 27∙1 | 30∙7 | 37∙0 |
| Diagnosed childhood ADHD (%) | | | | | | | | | |
| Yes (%) | 3∙6 | 3∙1 | 3∙8 | 7∙7 | 4∙0 | 4∙6 | 3∙9 | 5∙9 | 4∙4 |
| Maternal education level (%) | | | | | | | | | |
| <High school graduate (%) | 54∙7 | 57∙8 | 74∙9 | 79∙2 | 53∙3 | 53∙6 | 67∙3 | 57∙2 | 60∙2 |
| >=High school graduate (%) | 45∙3 | 42∙2 | 25∙1 | 20∙8 | 46∙7 | 46∙4 | 32∙7 | 42∙8 | 39∙8 |
| Paternal education level (%) | | | | | | | | | |
| <High school graduate (%) | 53∙2 | 60∙5 | 69∙4 | 74∙6 | 48∙6 | 53∙1 | 62∙5 | 67∙4 | 58∙3 |
| >=High school graduate (%) | 46∙8 | 39∙5 | 30∙6 | 25∙4 | 51∙4 | 46∙9 | 37∙5 | 42∙6 | 41∙7 |
| Household income per capita (monthly) (%) | | | | | | | | | |
| >=1500RMB (%) | 71∙0 | 69∙9 | 25∙5 | 25∙8 | 51∙6 | 37∙3 | 31∙4 | 36∙5 | 48∙4 |
| <1500RMB (%) | 29∙0 | 30∙1 | 74∙5 | 74∙2 | 48∙4 | 62∙7 | 68∙6 | 63∙5 | 51∙6 |
| Residential area per capita (m^2^) (%) | | | | | | | | | |
| <15 (%) | 10∙8 | 15∙3 | 22∙9 | 17∙3 | 9∙7 | 22∙9 | 11∙8 | 10∙7 | 14∙6 |
| 15-25 (%) | 24∙0 | 29∙1 | 39∙4 | 30∙7 | 28∙8 | 33∙7 | 35∙8 | 35∙8 | 30∙9 |
| 25-35 (%) | 25∙8 | 26∙6 | 20∙3 | 26∙8 | 29∙1 | 20∙3 | 28∙7 | 25∙2 | 25∙5 |
| >35 (%) | 39∙4 | 29∙0 | 17∙5 | 25∙5 | 32∙5 | 23∙2 | 23∙8 | 28∙3 | 29∙0 |
| Family size (persons) (%) | | | | | | | | | |
| 1-2 (%) | 5∙3 | 7∙4 | 6∙9 | 6∙1 | 7∙0 | 8∙0 | 7∙9 | 9∙0 | 7∙0 |
| 3 (%) | 53∙6 | 49∙4 | 48∙3 | 43∙6 | 52∙8 | 60∙6 | 64∙2 | 57∙5 | 53∙8 |
| 4-5 (%) | 34∙0 | 34∙5 | 37∙8 | 39∙6 | 31∙3 | 25∙4 | 9∙0 | 29∙2 | 32∙0 |
| >5 (%) | 7∙2 | 8∙7 | 7∙1 | 10∙8 | 8∙9 | 6∙0 | 57∙5 | 4∙3 | 7∙2 |
| Common cold (%) | | | | | | | | | |
| >5 times/year (%) | 20∙2 | 19∙9 | 13∙2 | 16∙3 | 19∙4 | 15∙1 | 10∙4 | 17∙4 | 17∙2 |
| <=5 times/year (%) | 79∙8 | 79∙1 | 86∙8 | 83∙7 | 70∙6 | 84∙9 | 89∙6 | 82∙6 | 82∙8 |
| Diagnosed recurrent otitis media (%) | | | | | | | | | |
| Yes (%) | 3∙8 | 5∙1 | 3∙0 | 3∙8 | 5∙2 | 4∙0 | 2∙6 | 2∙7 | 3∙9 |
| Snoring (>=2 nights/week in absence of common cold) (%) | | | | | | | | | |
| Yes (%) | 14∙8 | 14∙5 | 8∙2 | 14∙7 | 10∙9 | 10∙1 | 10∙3 | 10∙3 | 12∙2 |
| Sleep-disordered breathing (>=2 nights/week) (%) | | | | | | | | | |
| Yes (%) | 2∙6 | 2∙6 | 2∙1 | 4∙3 | 3∙1 | 3∙2 | 4∙1 | 3∙5 | 3∙1 |
| Paternal snoring (%) | | | | | | | | | |
| Yes (%) | 26∙5 | 28∙7 | 30∙4 | 34∙7 | 31∙4 | 32∙0 | 29∙9 | 36∙2 | 30∙6 |
| Maternal snoring (%) | | | | | | | | | |
| Yes (%) | 4∙1 | 5∙4 | 4∙4 | 7∙2 | 4∙5 | 4∙2 | 4∙6 | 5∙4 | 4∙8 |
| Paternal age at child’s birth (mean±SD) years | | | | | | | | | |
|  | 29∙3±4∙81 | 31∙5±4∙38 | 28∙3±4∙31 | 27∙7±4∙01 | 28∙4±4∙12 | 28∙1±3∙71 | 27∙9±4∙00 | 28∙8±3∙71 | 28∙9±4∙38 |
| Maternal age at child’s birth (mean±SD) years | | | | | | | | | |
|  | 26∙3±3∙98 | 28∙2±3∙79 | 25∙9±3∙53 | 25∙6±3∙51 | 26∙1±3∙25 | 25∙9±3∙17 | 26∙0±3∙56 | 26∙8±3∙31 | 26∙4±3∙65 |
